# Physician Incentives and Sex/Gender Differences in Depression Care: An Interrupted Time Series Analysis

**DOI:** 10.1089/heq.2019.0034

**Published:** 2020-03-13

**Authors:** Joseph H. Puyat, Arminee Kazanjian

**Affiliations:** ^1^ Centre for Health Evaluation and Outcome Sciences, Vancouver, Canada.; ^2^ School of Population and Public Health, University of British Columbia, Vancouver, Canada.

**Keywords:** health care disparities, mental health services, interrupted time series analysis, physician incentive plans

## Abstract

**Introduction:** Physician incentives have been shown to reduce socioeconomic disparities in health care. The impact on sex/gender inequalities, however, has rarely been investigated. Using population-based data, this study investigated sex/gender differences in depression care and the impact of physician incentives.

**Methods:** Deidentified health data from physician claims, hospitals, vital statistics, prescription database, and insurance plan registries in British Columbia, Canada, were examined, retrospectively. Individuals with depression were identified and their use of mental health services was tracked for 12 months following initial diagnosis. The following indicators were assessed: (1) counseling/psychotherapy (CP), (2) minimally adequate counseling/psychotherapy (MACP), (3) antidepressant therapy (AT), and (4) minimally adequate antidepressant therapy (MAAT). Sex/gender differences in these indicators before (January 2005–December 2007) and after (January 2008–December 2012) the introduction of physician incentives were estimated using interrupted time series analysis.

**Results:** Preintervention, the percentage of individuals with depression who received CP was higher among males (CP: 58.4%, MACP: 13.6%) than females (CP: 57.1%, MACP: 10.9%). In contrast, the percentage who received AT was higher among females (AT: 57.7%, MAAT: 47.4%) than males (AT: 53.6%, MAAT: 41.9%). These statistically significant sex/gender differences remain unchanged postintervention.

**Conclusions:** Sex/gender differences in depression care persist despite the introduction of physician incentives.

## Introduction

Financial incentives have long been used, often with modest results, to improve the quality of health care.^1–3^ An important component of health care quality is equity in access to and use of health services,^4,5^ although it is seldom examined as an end-point in many quality improvement initiatives that feature financial incentives as a core component.^6,7^ It is important to examine the impact of financial incentives primarily because of the huge public investments they entail,^8,9^ and also because of concerns that such interventions may exacerbate, rather than reduce existing health disparities.^10–12^

In previous studies, the disparities that have been examined in relation to the introduction of financial incentives include those based on age, sex, ethnicity, race, and socioeconomic status.^13–15^ These studies, mostly from the United Kingdom and the United States where financial incentives have been used extensively, showed that incentives have had modest or no impact at all on addressing existing disparities.^13–15^ Where it looked promising, the evidence points to a potential role for financial incentives in narrowing the gap between people residing in geographic areas that are low and high in measures of deprivation by facilitating increased use of health services in the latter group.^16^

In Canada, the use of financial incentives as a tool for improving mental health care was tested via a “natural experiment” in the province of British Columbia (BC). As part of its overall strategy to improve primary health care, BC deliberately avoided changing the way primary health care services are delivered, in direct contrast to what other Canadian provinces, such as Quebec and Ontario, have done.^17^ Instead of structural changes, BC rolled out a suite of financial incentives that target physicians who provide care to individuals with various health conditions, including mental health. Proponents of these operational enhancements believed that these will stem the decline in family practice that started in the mid-1990s and peaked in the mid-2000—a period marked by high levels of disenchantment among general practitioners (GPs) due to dissatisfaction with remuneration, workload complexity, and relationship with the provincial government.^17^

A study that examined specifically the impact of introducing physician incentives in BC on mental health services delivered in primary care settings has shown that providing physician incentives can change the pattern of access to depression care at the population level, although the magnitude of the estimated impact was modest.^18^ The study found that the proportion of individuals who accessed psychological therapy increased, while the proportion of those who filled antidepressant prescriptions declined over the study period. The study also found that physician continuity of care, an indicator that measures whether patients see the same physician for outpatient care, ceased to decline after the incentives were introduced.^18^

Whether the introduction of physician incentives in BC has changed disparities in the use of mental health care has never been examined. Among the health inequities that can be examined are sex/gender disparities, which need to be examined given the robust association between gender and mental health. Of particular interest is depression, which has higher incidence and prevalence among women than men.^19^ Sex/gender differences in the use of effective treatments for depression (i.e., psychological and antidepressant therapies) signal potential health inequities since existing evidence indicates that these treatment modalities are equally effective for both sexes/genders.^20–22^ Likewise, health inequities potentially exist if differences in patterns of use are incongruent with treatment preference, such as when women, for example, are found to be less likely than men to receive psychological therapy when most women actually prefer receiving psychological therapy to treat depression.^23^

In this study, we investigated the impact of financial incentives on sex/gender disparities in depression care, using indicators that measure receipt of counseling/psychotherapy (CP); minimally adequate counseling/psychotherapy (MACP), defined as ≥4 sessions of CP; antidepressant therapy (AT); and minimally adequate antidepressant therapy (MAAT), defined as ≥84 days of AT.

## Methods

### Data

We examined deidentified and individual-level health data from virtually everyone in BC, excluding a small percentage (4%) of individuals whose health care is covered under federal jurisdiction (i.e., registered status Indians/aboriginals, veterans, federal penitentiary inmates, and members of the Royal Canadian Mounted Police). The databases we analyzed include the government-sponsored provincial health insurance registry, physician claims database, hospital database, outpatient prescription database, and the provincial death registry. A common study identifier generated by Population Data BC linked all these databases (Table 1). Permission to access data was provided by the BC Ministry of Health and the BC College of Pharmacists. The Behavioral Research Ethics Board of the University of British Columbia granted ethics approval for the study (UBC BREB No. H14-00847).

**Table 1.** Data Sources and Data Fields Used in the Study

Database and source	Data fields
*Consolidation File, 2004–2013*	Patient study id, birth month, Birth year, sex, 3-digit postal code (or forward sortation address), neighborhood income quintile, number of days registered in the provincial health services plan
BC Ministry of Health [creator] (2014): Consolidation File (MSP Registration & Premium Billing). V2. Population Data BC [publisher]. Data Extract. Ministry of Health (2014). www.popdata.bc.ca/data	
*Physician Claims Database, 2004–2013*	Patient study id, date service was provided, practitioner study ID number, specialty code, service code, fee item codes, service units, amount paid, service units, ICD9 diagnostic codes
BC Ministry of Health (2015): Medical Services Plan (MSP) Payment Information File. V2. Population Data BC [publisher]. Data Extract. MOH (2014). www.popdata.bc.ca/data	
*Hospital Separations, 2004–2013*	Patient study id, discharge or separation date, and ICD10 diagnostic codes
Canadian Institutes for Health Information [creator] (2015): Discharge Abstract Database (Hospital Separations). V2. Population Data BC [publisher]. Data Extract. MOH (2014). www.popdata.bc.ca/data	
*Prescription Database, 2004–2013*	Patient study id, drug identification number, date dispensed, quantity dispensed, and day's supply
BC Ministry of Health [creator] (2014): PharmaNet. V2. BC Ministry of Health [publisher]. Data Extract. Data Stewardship Committee (2014). www.popdata.bc.ca/data	
*Deaths Registry, 2004–2013*	Patient study id, year and month of death
BC Vital Statistics Agency [creator] (2014): Vital Statistics Deaths. V2. Population Data BC. Data Extract BC Vital Statistics Agency (2014). www.popdata.bc.ca/data	

BC, British Columbia.

### Study cohort

We identified cohorts of males and females, older than 18 years, who received new major depressive disorder diagnoses in each of the months between January 2005 and December 2012. Depression diagnoses were ascertained through the primary diagnoses associated with a physician visit or hospital discharge. This is a valid method of identifying depression cases, especially when used in a population-level analysis.^24,25^ Details of how the monthly cohorts were derived from the linked data have been previously described.^18,26^

### Outcome variables

To investigate changes in mental health service use over a period of 8 years, we examined receipt of the following: (1) one or more sessions of counseling or psychotherapy; (2) MACP, defined as 4 or more CP sessions; (3) one or more filled prescriptions for an antidepressant; and (4) minimally adequate AT, defined as 84 or more days of AT. Criteria for these indicators were deemed met if services were received within 12 months following initial diagnosis of depression.^18^

### Explanatory variables

A primary variable of interest in this study is sex/gender. Currently, there are no definitive and universally accepted definitions of “gender” or “sex.”^27^ Gender is typically regarded as a social construct defined by the roles, relationships, and behaviors of women and men, whereas sex is considered to be a physical construct linked to the biology or physiology of females and males.^27^ Sex and gender are intricately and significantly related, and both are important when studying health outcomes.^28^ In this study, we used the sex field (male/female) of the linked health administrative databases as a construct that captures aspects of both sex and gender.^28–30^ We used the term sex/gender throughout the article to underscore the large overlap between the two constructs and to indicate that the disparities we studied cannot be attributed solely to sex.

Another explanatory variable of interest is the introduction of physician incentives, which putatively influences population-level changes in the use of mental health services. Known as the Mental Health Initiative,^31^ the policy was introduced in BC in January 1, 2008, with an initial budget of $8 million.^32^ The overall aim of the policy was to remove the financial barriers that family physicians or GPs experience when managing patients with mental health issues.^33^ The financial incentives were embedded in a revised fee-for-service schedule, allowing GPs who prepare comprehensive treatment plans to receive compensation for being a patient's major source of care. The revised fee schedule increased the number of billable CP sessions from 4 to 8 sessions per year, and introduced fee codes for coordinating with other health care providers or for conducting follow-ups by e-mail or telephone.^31^

### Analysis

We constructed monthly indicators for each of the four measures. Values for these indicators were calculated by dividing the total count of individuals who met criteria for an indicator in a given month by the total number of individuals with new diagnoses of depression in the same month, multiplied by 100.

$$\begin{array}{c} \%Minimallyadequate\\ antidepressanttherapy\\ inJanuary2008 \end{array}=\frac{\begin{array}{c} Countofindividualsfromthedenominator\\ whofilledantidepressantsfor\geq 84days\\ within12monthsofinitialdiagnosis \end{array}}{\begin{array}{c} Totalcountofindividuals\\ diagnosedwithdepression\\ betweenJanuary1and31,2008 \end{array}}\times 100$$

To estimate the changes in the levels and trend before and after the intervention, we used ordinary least squares regression with Newey–West standard errors to account for potential autocorrelation.^34^ The regression model has the following general form:

$$\begin{array}{cc} {Ŷ}_{t}= & \beta_{0}+\beta_{1}month_{t}+\beta_{2}intervention_{t}\\ & +\beta_{3}intervention_{t}Xmonth_{t}+\beta_{4}males\\ & +\beta_{5}menXmonth_{t}+\beta_{6}menXintervention_{t}\\ & +\beta_{7}menXintervention_{t}Xmonth_{t}+e_{t}, \end{array}$$

where ${Ŷ}_{t}$ is the average level of an indicator at month *t*; $\beta_{0}$, the intercept; $\beta_{1}$, the monthly rate of increase (or trend) among females; $\beta_{2}$, the change in the level after the intervention as of January 2008 among females; $\beta_{3}$, the change in the trend after the intervention among females; $\beta_{4}$, the difference between females and males in the level before the intervention; $\beta_{5}$, the difference in the trend between females and males before the intervention; $\beta_{6}$, the difference between females and males in the postintervention change in the levels as of January 2008; $\beta_{7}$, the difference between females and males in the postintervention change in the trend; and *e_t_*, the residual. Of interest are the magnitude and direction of $\beta_{6}$ and $\beta_{7}$, which measure the intervention's immediate and longer term impact on sex/gender differences. Large, negative, and statistically significant values for these two coefficients are strong indications that disparities have decreased immediately and continue to decrease after the intervention. We also postestimated the magnitude of three other relevant quantities: sex/gender differences before (average female–male differences, preintervention), sex/gender differences after (average female–male differences, postintervention), and the change in sex/gender differences before and after the intervention (pre/post change in the average female/male differences).

To facilitate understanding of the sex/gender disparities and any potential postintervention changes that occur either immediately or over time, we complemented our analyses with figures. For each indicator, we plotted data points, superimposed with separate regression lines for females and males. In these figures, distinct and clear separation in data points and regression lines between females and males are indicative of sex/gender differences. Parallel lines suggest persistent differences, while converging or diverging lines suggest decreasing or increasing differences.

We used SAS/SQL software version 9.4 to extract, link, and manage the multiyear data from multiple databases; Stata version 14.2 to run the regression analyses and obtain postestimation values; and R version 3.4.1 to generate the plots.

## Results

During the study period, the mean number of individuals diagnosed with depression each year was 106,277 (standard deviation [SD]=5027; min=97,760; max=112,458). On average, about 65% of those diagnosed with depression were females (mean=69,529; SD=2447; min=65,070; max=72,723), while 35% were males (mean=36,749; SD=2744; min=32,690; max=39,735).

### Counseling/psychotherapy

Sex/gender disparity in the receipt of CP was present and unaffected by the introduction of physician incentives. Results from the interrupted time series analysis did not show evidence of postintervention change in sex/gender disparities, either immediately ($\beta_{6}$=0.02, 95% confidence interval [CI]=−1.41 to 1.45) or over time ($\beta_{7}$=0.00, 95% CI=−0.05 to 0.04). Similarly, the average sex/gender disparity in the receipt of CP before the intervention was 1.33 (95% CI=0.86 to 1.80); postintervention it was 1.35 (95% CI=0.86 to 1.80), resulting in a pre/post change of −0.02 (95% CI=−0.64 to 0.60) (Table 2).

**Table 2.** Model Estimates of the Impact of Physician Incentives on Sex/Gender Disparities in Depression Care

	Counseling or psychotherapy					Antidepressant therapy		
	One or more		Minimally adequate		One or more		Minimally adequate	
	Estimate	95% CI	Estimate	95% CI	Estimate	95% CI	Estimate	95% CI
Model estimates								
$\beta_{0}$	57.14	56.80, 57.49	10.93	10.66, 11.19	57.71	57.13, 58.29	47.43	46.67, 48.19
$\beta_{1}$	−0.04	−0.06, −0.02	0.02	0.01, 0.03	0.09	0.06, 0.11	0.08	0.05, 0.12
$\beta_{2}$	1.36	0.67, 2.05	−0.03	−0.47, 0.41	−1.71	−2.54, −0.87	−1.91	−2.75, −1.07
$\beta_{3}$	0.05	0.03, 0.07	0.02	0.00, 0.03	−0.06	−0.09, −0.03	−0.03	−0.06, 0.01
$\beta_{4}$	1.28	0.65, 1.91	2.66	2.31, 3.02	−4.07	−4.95, −3.19	−5.51	−6.49, −4.52
$\beta_{5}$	0.00	−0.04, 0.04	−0.01	−0.03, 0.00	0.00	−0.03, 0.04	0.00	−0.04, 0.04
$\beta_{6}$	0.02	−1.41, 1.45	0.44	−0.21, 1.09	0.53	−0.47, 1.53	0.28	−0.84, 1.41
$\beta_{7}$	0.00	−0.05, 0.04	0.01	−0.02, 0.03	−0.02	−0.06, 0.02	−0.01	−0.06, 0.04
Preintervention								
Mean level in January 2005, females	57.10	56.77, 57.44	10.95	10.69, 11.20	57.80	57.24, 58.35	47.52	46.79, 48.25
Mean level in January 2005, males	58.38	57.89, 58.88	13.60	13.37, 13.82	53.73	53.09, 54.37	42.01	41.41, 42.61
Slope, females	−0.04	−0.06, −0.02	0.02	0.01, 0.03	0.09	0.06, 0.11	0.08	0.05, 0.12
Slope, males	−0.04	−0.07, 0.00	0.00	−0.01, 0.02	0.09	0.06, 0.12	0.09	0.06, 0.11
Average female/male differences	1.33	0.86, 1.80	2.41	2.17, 2.65	−4.03	−4.39, −3.67	−5.50	−5.96, −5.04
Postintervention								
Mean level in January 2008, females	57.05	56.55, 57.56	11.58	11.22, 11.94	59.25	58.51, 59.99	48.66	48.03, 49.29
Mean level in January 2008, males	58.46	57.95, 58.96	14.17	13.88, 14.47	55.79	55.37, 56.21	43.45	42.95, 43.96
Slope, females	0.01	−0.01, 0.02	0.04	0.03, 0.05	0.03	0.01, 0.05	0.06	0.04, 0.07
Slope, males	0.00	−0.01, 0.02	0.03	0.02, 0.04	0.01	0.00, 0.02	0.05	0.03, 0.06
Average female/male differences	1.35	0.98, 1.73	2.36	2.14, 2.58	−4.02	−4.42, −3.63	−5.58	−5.97, −5.19
Pre/post changes								
Change in level, females	1.36	0.67, 2.05	−0.03	−0.47, 0.41	−1.71	−2.54, −0.87	−1.91	−2.75, −1.07
Change in level, males	1.38	0.13, 2.63	0.41	−0.06, 0.89	−1.18	−1.72, −0.64	−1.62	−2.37, −0.88
Female/male differences in change in level	0.02	−1.41, 1.45	0.44	−0.21, 1.09	0.53	−0.47, 1.53	0.28	−0.84, 1.41
Change in slope, females	0.05	0.03, 0.07	0.02	0.00, 0.03	−0.06	−0.09, −0.03	−0.03	−0.06, 0.01
Change in slope, males	0.04	0.00, 0.08	0.03	0.01, 0.04	−0.08	−0.11, −0.05	−0.04	−0.07, −0.01
Female/male differences in change in slope	0.00	−0.05, 0.04	0.01	−0.02, 0.03	−0.02	−0.06, 0.02	−0.01	−0.06, 0.04
Change in average female/male differences	−0.02	−0.64, 0.60	0.05	−0.28, 0.38	−0.01	−0.54, 0.52	0.08	−0.52, 0.67

The reported values represent percentages and 95% CIs. The regression formula with Newey–West standard errors is:

$$\begin{array}{c} {Ŷ}_{t}=\beta_{0}+\beta_{1}month_{t}+\beta_{2}intervention_{t}+\beta_{3}intervention_{t}Xmonth_{t}+\beta_{4}males+\beta_{5}malesXmonth_{t}\\ +\beta_{6}malesXintervention_{t}+\beta_{7}malesXintervention_{t}Xmonth_{t}+e_{t}, \end{array}$$

where ${Ŷ}_{t}$ is the level of the indicator at a given month; $\beta_{0}$, the intercept; $\beta_{1}$, the monthly rate of increase (trend) among females; $\beta_{2}$, the change in the level after the intervention in January 2008, among females; $\beta_{3}$, the change in the trend after the intervention, among females; $\beta_{4}$, the difference between females and males in the level before the intervention; $\beta_{5}$, the difference in the trend between females and males before the intervention; $\beta_{6}$, the difference between females and males in the postintervention change in the levels as of January 2008; $\beta_{7}$, the difference between females and males in the postintervention change in the trend; and *e_t_*, the residual. Females were coded as 0 and males were coded as 1.

CI, confidence interval.

**FIG. 1.**
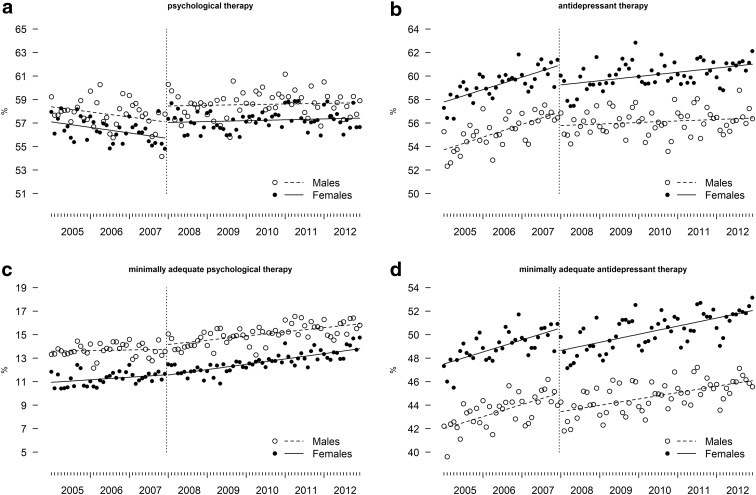
Sex/gender differences in **(a)** psychological therapy, **(b)** AT, **(c)** minimally adequate psychological therapy, and **(d)** minimally adequate AT, before (2005–2007) and after (2008–2012) the introduction of physician incentives in British Columbia, Canada. AT, antidepressant therapy.

Figure 1a shows that the proportion of those who received at least one CP was higher among males than females in most of the months before the introduction of the intervention. There was also a decreasing trend in both gender groups, which was disrupted by the intervention. Despite the disruption, however, the parallel regression lines, representing the predicted average for males and females postintervention, indicate that the intervention had no impact on the prevailing disparities in the receipt of CP.

### Minimally adequate counseling/psychotherapy

There was no evidence that the introduction of physician incentives changed the existing gender disparity (i.e., favoring males) in the receipt of CP. No meaningful change occurred immediately ($\beta_{6}$=0.44, 95% CI=−0.21 to 1.09) or over time ($\beta_{6}$=0.01, 95% CI=−0.02 to 0.03). The gender differences in the receipt of MACP pre- and postintervention were 2.41 (95% CI=2.17 to 2.65) and 2.36 (95% CI=2.14 to 2.58), with a pre/post estimated change of 0.05 (95% CI=−0.28 to 0.38) (Table 2).

Compared with Figure 1a, the plot for this indicator (Fig. 1c) clearly showed a higher proportion of males receiving MACP throughout the study period. Preintervention there was a slight increasing trend for females but not males. Postintervention the trend was virtually the same for both groups. Overall, the parallel regression lines throughout the study period indicate the intervention's lack of impact on the existing disparities in the receipt of MACP.

### Antidepressant therapy

The magnitude of the sex/gender disparity in the receipt of AT was greater than the sex/gender disparity in the receipt of counseling or psychotherapy. The results also did not indicate any meaningful change in disparity that occurred immediately ($\beta_{6}$=0.53, 95% CI=−0.47 to 1.53) or over time ($\beta_{7}$=−0.02, 95% CI=−0.06 to 0.02). The average gender disparities in the receipt of antidepressant pre- and postintervention were −4.03 (95% CI=−4.39 to −3.67) and −4.02 (95% CI=−4.42 to −3.63). The estimated pre/post change was −0.01 (95% CI=−0.54 to 0.52) (Table 2).

Figure 1b shows that throughout the entire study period, the proportion of those who filled at least one prescription for antidepressants was higher among females than males. Postintervention the levels have decreased appreciably and the upward trend slackened. However, these changes occurred with about the same magnitude in both groups. The disparity in the receipt of AT between females and males remained virtually unaffected by the intervention.

### Minimally adequate antidepressant therapy

Similar to the indicator for the receipt of AT, there was no evidence of postintervention change in the disparities between females and males in the receipt of MACP. Specifically, there were no meaningful changes in the magnitude of the disparity that occurred immediately ($\beta_{6}$=0.28, 95% CI=−0.84 to 1.41) or over time ($\beta_{6}$=−0.01, 95% CI=−0.06 to 0.04). Likewise, the preintervention average difference of −5.50 (95% CI=−5.96 to −5.04) between females and males was not substantially different from the postintervention average difference of −5.58 (95% CI=−5.97 to −5.19) (Table 2).

Compared with Figure 1b, d shows a clearer and wider separation of the data points and regression lines that represent the proportion of females and males who receive MAAT. These suggest that the magnitude of gender disparity associated with receipt of MAAT is slightly greater than that found in the receipt of AT. Virtually identical changes in females and males, with respect to the postintervention levels and monthly trend, suggest that the impact of the intervention on gender disparity in the receipt of MAAT has largely been inconsequential.

## Discussion

We sought to determine and estimate the impact of physician incentives on sex/gender differences in the receipt of psychological and antidepressant therapies. Our results show that although modest changes in the pattern of use for psychological and AT have occurred after physician incentives were introduced in 2008 in BC, sex/gender disparities in the use of these therapies were hardly impacted by the incentives. The proportion of those with depression who receive psychological therapy remained higher in males than females, while the proportion that receives antidepressants remained higher in females than males.

As this is the first study we know of that specifically examined the impact of financial incentives on sex/gender differences in depression care, direct comparison with previous findings is not possible. Generally, studies that assessed the impact of physician incentives on other sex/gender-based health inequities are scarce and the few that are published suggest no consistent pattern of impact.^13^

In this study, we did not observe any significant exacerbation of sex/gender differences in the use of depression care after physician incentives were introduced. In previous studies, physician incentives have been reported to have the unintended consequence of increasing sex/gender-based health inequities. For example, the introduction of physician incentives under U.K.'s Quality and Outcomes Framework (QOF) in 2004 has been shown to result in women being less likely than men to be included in the recording of quality indicators for coronary heart disease^35^ and diabetes.^36^

While it may be argued that the physician incentives implemented in BC were not specifically intended to address health inequities, it would not be entirely unrealistic to expect physician incentives to have measurable effects toward reducing sex differences in depression care. Under certain circumstances, financial incentives can have a meaningful impact on reducing sex/gender-based health inequities, as shown, for example, in QOF studies that examined the impact of financial incentives on sex/gender differences in a number of care processes for stroke/transient ischemic attack,^37^ and in indicators of success for smoking cessation/prevention programs.^38^

A number of recommendations in the literature have been offered to help ensure that substantial public investments, such as physician incentives, contribute to better health care not just for the general population but also across subpopulations. It has been emphasized, for instance, the importance of not assuming that financial incentives intended to improve the quality of care overall will automatically result in equal benefits for everyone.^39^ Health inequities can be impervious to the effects of financial incentives, as findings from this study illustrate. In some cases, financial incentives can exacerbate existing health inequities.^7,35,36^ Another recommendation is to include specific targets for reducing disparities, which may require collecting data stratified by subpopulations and reporting robust health inequity measures.^6,39^ Last, financial incentives could be designed to address patient-, provider-, health system-, and population-level factors that promote and sustain various types of health inequities.^6^ In the case of sex/gender-based health inequalities, this requires a concerted, deliberate, and systematic approach to understanding the underlying processes or pathways that give rise to these disparities.^40^

A few points warrant consideration when interpreting the study findings. First, our data on depression care represent services that are covered by public health insurance. Second, we were unable to examine the appropriateness of CP provided by physicians due to lack of relevant data. Third, the results regarding the use of AT were based on records of prescription fills, which can underestimate prescribing rates or overestimate actual use. Last, we were limited to using sex as a variable for assessing sex/gender differences because sex is the closest representation of gender that we can derive from health administrative data. As a consequence, our analyses considered gender as a dichotomous variable, even though it is more appropriately examined as a spectrum^41^ particularly in the context of health care inequalities.

## Abbreviations Used

AT: antidepressant therapy
BC: British Columbia
CI: confidence interval
CP: counseling/psychotherapy
GPs: general practitioners
MAAT: minimally adequate antidepressant therapy
MACP: minimally adequate counseling/psychotherapy
QOF: Quality and Outcomes Framework
SD: standard deviation

## References

[1] Langdown C, Peckham S The use of financial incentives to help improve health outcomes: is the quality and outcomes framework fit for purpose? A systematic review. J Public Health. 2014;36:251–258 10.1093/pubmed/fdt077 23929885

[2] Eijkenaar F, Emmert M, Scheppach M, et al. Effects of pay for performance in health care: a systematic review of systematic reviews. Health Policy. 2013;110:115–130 23380190 10.1016/j.healthpol.2013.01.008

[3] Scott A, Sivey P, Ait Ouakrim D, et al. The effect of financial incentives on the quality of health care provided by primary care physicians. Cochrane Database Syst Rev. 2011;CD008451 21901722 10.1002/14651858.CD008451.pub2

[4] Leatherman ST, Sutherland K, Canadian Health Services Research Foundation, et al. Quality of Healthcare in Canada: A Chartbook. Ottawa, Ontario: Canadian Health Services Research Foundation 2010 Available at www.deslibris.ca/ID/221531 Accessed 49, 2018

[5] Crossing the Quality Chasm: A New Health System for the 21st Century. 2001 Available at www.iom.edu/Reports/2001/Crossing-the-Quality-Chasm-A-New-Health-System-for-the-21st-Century.aspx Accessed 44, 2014 25057539

[6] Chin MH. Creating the business case for achieving health equity. J Gen Intern Med. 2016;31:792–796 26883523 10.1007/s11606-016-3604-7 PMC4907942

[7] Chien AT, Chin MH, Davis AM, et al. Pay for performance, public reporting, and racial disparities in health care. Med Care Res Rev. 2007;64:283S–304S 17881629 10.1177/1077558707305426

[8] MacCarthy D, Hollander MJ RISQy business (relationships, incentives, supports, and quality): evolution of the British Columbia model of primary care (patient-centered medical home). Perm J. 2014;18:43–48 10.7812/TPP/13-083 PMC4022557 24867550

[9] Timmins N. Do GPs deserve their recent pay rise? BMJ. 2005;331:800 10.1136/bmj.331.7520.800 PMC1246073 16210280

[10] Karve AM, Ou F-S, Lytle BL, et al. Potential unintended financial consequences of pay-for-performance on the quality of care for minority patients. Am Heart J. 2008;155:571–576 18294498 10.1016/j.ahj.2007.10.043

[11] Hood RG. Pay-for-performance—financial health disparities and the impact on healthcare disparities. J Natl Med Assoc. 2007;99:6 PMC2574309 17722677

[12] Casalino LP, Elster A, Eisenberg A, et al. Will pay-for-performance and quality reporting affect health care disparities? Health Aff (Millwood). 2007;26:w405–w414 17426053 10.1377/hlthaff.26.3.w405

[13] Boeckxstaens P, Smedt DD, Maeseneer JD, et al. The equity dimension in evaluations of the quality and outcomes framework: a systematic review. BMC Health Serv Res. 2011;11:209 21880136 10.1186/1472-6963-11-209 PMC3182892

[14] Tao W, Agerholm J, Burström B The impact of reimbursement systems on equity in access and quality of primary care: a systematic literature review. BMC Health Serv Res 2016;16:542 27716250 10.1186/s12913-016-1805-8 PMC5050924

[15] Alshamsan R, Majeed A, Ashworth M, et al. Impact of pay for performance on inequalities in health care: systematic review. J Health Serv Res Policy. 2010;15:178–184 20555042 10.1258/jhsrp.2010.009113

[16] Doran T, Fullwood C, Kontopantelis E, et al. Effect of financial incentives on inequalities in the delivery of primary clinical care in England: analysis of clinical activity indicators for the quality and outcomes framework. Lancet. 2008;372:728–736 18701159 10.1016/S0140-6736(08)61123-X

[17] Tregillus VHF, Cavers WJR General Practice Services Committee: improving primary care for BC physicians and patients. Healthc Q. 2011;14:1–6

[18] Puyat JH, Kazanjian A, Wong H, et al. Is the road to mental health paved with good incentives? Estimating the population impact of physician incentives on mental health care using linked administrative data. Med Care. 2017;55:182–190 27632766 10.1097/MLR.0000000000000639

[19] Kuehner C. Why is depression more common among women than among men? Lancet Psychiatry. 2017;4:146–158 27856392 10.1016/S2215-0366(16)30263-2

[20] Cuijpers P, Weitz E, Twisk J, et al. Gender as predictor and moderator of outcome in cognitive behavior therapy and pharmacotherapy for adult depression: an ‘individual patient data’ meta-analysis. Depress Anxiety. 2014;31:941–951 25407584 10.1002/da.22328

[21] Weissman MM. Treatment of depression: men and women are different? Am J Psychiatry. 2014;171:384–387 24687191 10.1176/appi.ajp.2013.13121668

[22] Parikh SV, Quilty LC, Ravitz P, et al. Canadian Network for Mood and Anxiety Treatments (CANMAT) 2016 clinical guidelines for the management of adults with major depressive disorder: section 2. Psychological treatments. Can J Psychiatry. 2016;61:524–539 27486150 10.1177/0706743716659418 PMC4994791

[23] McHugh RK, Whitton SW, Peckham AD, et al. Patient preference for psychological vs pharmacologic treatment of psychiatric disorders: a meta-analytic review. J Clin Psychiatry. 2013;74:595–602 23842011 10.4088/JCP.12r07757 PMC4156137

[24] Fiest KM, Jette N, Quan H, et al. Systematic review and assessment of validated case definitions for depression in administrative data. BMC Psychiatry. 2014;14:289 25322690 10.1186/s12888-014-0289-5 PMC4201696

[25] Morrison K, Feng F, Puyat JH, et al. Validation of Chronic Disease Canadian Primary Care Sentinel Surveillance Database in BC. Poster Presented at: 2013 Family Medicine Forum. Vancouver, BC: College of Family Physicians of Canada, 2013

[26] Puyat JH, Kazanjian A, Goldner EM, et al. How often do individuals with major depression receive minimally adequate treatment? A population-based, data linkage study. Can J Psychiatry. 2016;61:394–404

[27] Getz L. Sex, Gender and Health Research Guide: a Tool for CIHR Applicants. 2016. Available at www.genderportal.eu/resources/sex-gender-and-health-research-guide-tool-cihr-applicants Accessed 831, 2018

[28] Krieger N. Genders, sexes, and health: what are the connections—and why does it matter? Int J Epidemiol. 2003;32:652–657 12913047 10.1093/ije/dyg156

[29] Fausto-Sterling A. *Sex/Gender: Biology in a Social World*. 1st ed. New York: Routledge, 2012

[30] Springer KW, Mager Stellman J, Jordan-Young RM Beyond a catalogue of differences: a theoretical frame and good practice guidelines for researching sex/gender in human health. Soc Sci Med. 2012;74:1817–1824 21724313 10.1016/j.socscimed.2011.05.033

[31] Cavers B. Community-based mental health initiative: patients and GPs to benefit. Br Columbia Med J. 2008;50:63

[32] BC Ministry of Health, GPSC. General Practice Services Committee Annual Report 2007–2008. 2008 Available at www.health.gov.bc.ca/library/publications/year/2008/GPSC_AnnualReport0708.pdf Accessed 1222, 2013

[33] GPSC. Mental Health Fees Summary Information. Pre-reading for Practice Support Program Mental Health—Making it Real. Available at www.gpscbc.ca/system/files/MH_fee_codes_summary.pdf Accessed 519, 2009

[34] Newey WK, West KD A simple, positive semi-definite, heteroskedasticity and autocorrelation consistent covariance matrix. Econometrica. 1987;55:703–708.

[35] McGovern MP, Boroujerdi MA, Taylor MW, et al. The effect of the UK incentive-based contract on the management of patients with coronary heart disease in primary care. Fam Pract. 2008;25:33–39 18222938 10.1093/fampra/cmm073

[36] McGovern MP, Williams DJ, Hannaford PC, et al. Introduction of a new incentive and target-based contract for family physicians in the UK: good for older patients with diabetes but less good for women? Diabet Med. 2008;25:1083–1089 18937676 10.1111/j.1464-5491.2008.02544.x

[37] Simpson CR, Hannaford PC, Lefevre K, et al. Effect of the UK incentive-based contract on the management of patients with stroke in primary care. Stroke. 2006;37:2354–2360 16873713 10.1161/01.STR.0000236067.37267.88

[38] Millett C, Gray J, Saxena S, et al. Impact of a pay-for-performance incentive on support for smoking cessation and on smoking prevalence among people with diabetes. CMAJ. 2007;176:1705–1710 17548383 10.1503/cmaj.061556 PMC1877840

[39] James CV, Rosenbaum S Paying for quality care: implications for racial and ethnic health disparities in pediatric asthma. Pediatrics. 2009;123 S205–S210 19221165 10.1542/peds.2008-2233L

[40] Bierman AS. Sex matters: gender disparities in quality and outcomes of care. Can Med Assoc J. 2007;177:1520–1521 18003953 10.1503/cmaj.071541 PMC2096490

[41] Johnson JL, Repta R. Sex and gender: beyond the binaries. In: Oliffe JL, Greaves L (eds.). Designing and Conducting Gender, Sex, and Health Research. Thousand Oaks, CA: SAGE Publications, 2012, pp. 17–37

